# Adolescent pregnancy and transition to adulthood in young users of the SUS

**DOI:** 10.1590/S1518-8787.2017051006528

**Published:** 2017-03-31

**Authors:** Elisabeth Meloni Vieira, Aylene Bousquat, Claudia Renata dos Santos Barros, Maria Cecilia Goi Porto Alves

**Affiliations:** I Departamento de Medicina Social. Faculdade de Medicina. Universidade de São Paulo. Ribeirão Preto, SP, Brasil; II Departamento de Política, Gestão e Saúde. Faculdade de Saúde Pública. Universidade de São Paulo. São Paulo, SP, Brasil; III Programa de Pós-Graduação em Saúde Coletiva. Universidade Católica de Santos. Santos, SP, Brasil; IV Instituto de Saúde. Secretaria de Estado da Saúde de São Paulo. São Paulo, SP, Brasil

**Keywords:** Pregnancy in Adolescence, Young Adult, Gender and Health, Social Support

## Abstract

**OBJECTIVE:**

The objective of this study is to contextualize adolescent pregnancy from milestones associated with the process of transition from youth to adulthood.

**METHODS:**

This is a cross-sectional study conducted with 200 adolescents, users of the Brazilian Unified Health System. The sample size for the estimation of proportions has been calculated assuming a population ratio of 0.50 and 95% confidence level. The dependent variables – planned pregnancy, living with a partner, and having left the parents’ house – have been considered as markers of transition from dependence to independence, from youth to adulthood. In the analysis of the associated factors, we have used the Poisson model with robust variance.

**RESULTS:**

Average age was 17.3 years, and most adolescents lived with a partner; approximately half of the adolescents got pregnant from their first partner and the average age of first sexual intercourse was 14.6 years. Only 19% of the adolescents were studying and most dropped out of school before the beginning of the pregnancy. In the bivariate and multiple analysis, we could see that the relationship with a partner for more than two years was associated with the three dependent variables.

**CONCLUSIONS:**

The path of transition to adulthood has been the establishment of a link with a partner and consequent pregnancy, suggesting a clear pattern of male guardianship. The changing role of women in society observed in recent decades, which means choosing a professional career, defining the number of children, and choosing their partner(s), has not reached these young persons.

## RESUMO

**OBJETIVO:**

Contextualizar a gestação em adolescentes a partir de marcos associados ao processo de transição da juventude para a vida adulta.

**MÉTODOS:**

Estudo transversal realizado com 200 adolescentes usuárias do Sistema Único de Saúde. O tamanho da amostra para a estimação de proporções foi calculado considerando uma proporção populacional de 0,50, e nível de confiança de 95%. As variáveis dependentes – gestação planejada, morar com o parceiro e ter saído da casa dos pais – foram consideradas marcadores da transição da dependência para a independência, da juventude para a vida adulta. Na análise dos fatores associados, utilizou-se o modelo de Poisson com variância robusta.

**RESULTADOS:**

A idade média foi 17,3 anos, a maioria coabitava com o companheiro; aproximadamente metade engravidou do primeiro parceiro e a idade média da primeira relação sexual foi 14,6 anos. Apenas 19% das jovens estudavam e o abandono escolar foi, na maior parte, anterior ao início da gestação. Nas análises bivariadas e na análise múltipla, observou-se que relacionar-se com o parceiro há mais de dois anos se associou às três variáveis dependentes.

**CONCLUSÕES:**

O caminho de transição para a vida adulta foi o estabelecimento de um vínculo com um parceiro e consequente maternidade, sugerindo padrão claro de tutela masculina. A mudança do papel da mulher na sociedade observada nos últimas décadas, que implica optar por uma carreira profissional, definir número de filhos e escolher o(s) parceiro(s),não chegou a parcela dessas jovens.

## INTRODUCTION

Adolescent pregnancy is often addressed as a single phenomenon, uniform, and almost timeless^8^; an early event associated with the poorest and least educated layers of the population^20^. This homogenization prevents us from understanding the many realities and differences experienced by young mothers^20^. Central issues in the lives of these young persons, such as the desire to become pregnant, the constitution of nuclear families, and the change in their social status are often disregarded^25^.

In Brazil, the fertility of young women aged between 15 and 19 years grew until the end of the 20th century, and began declining in the early years of the 21st century. However, even with this recent fall, we can affirm that there is a rejuvenation of the fertility in the country^4^. In absolute terms, 559,991 births were recorded for mothers under the age of 19 years in 2013, a magnitude that reinforces the importance of studies on the subject.

Adolescence, which is a new and modern age between childhood and adulthood, is a time of transition, in which the progression from dependence to independence is expected in relation to the family of origin. The first studies focused on the subject were based on the idea that this transition would be procedural and could be confirmed from the milestones of life stories^12^, which would occur sequentially and unidirectionally (end of studies, entry into the labor market, leaving of the parents’ house, marriage, first child). Several authors^7^ ^,^ ^13^ consider the birth of the first child to be the final stage of this transition. This linear conception of transition was based on the reality of central capitalist countries after World War II, a period marked by high economic growth and great job offer^11^.

However, this sequential and unidirectional approach related to the transition to adulthood has been considered as insufficient to respond to the complexity of the social and economic relations present in contemporary societies^11^ ^,^ ^26^. Camarano and Mello^11^ indicate two orders of factors involved in the change in the process of transition. On the one hand, we have increased schooling and difficulties of entry into the labor market; on the other hand, we have changes in the sexual pattern and separation of sexual activity, marital union, and parenting.

Galland^17^ highlights that a contemporary characteristic in the process of transition to adulthood is the reversibility and the interweaving between these milestones, resulting from changes in the structure of the world of work and the new intergenerational relations. Thus, we can identify new consequences from the milestones of the transition to adulthood, among which the birth of the first child, in isolation, would not indicate the end of the transition by itself.

Analyzing the Brazilian reality, Camarano and Mello^11^ demonstrate a new configuration for this transition, in which sequential transition patterns coexist with different ways that mark the entry into adulthood. We can say that different processes of transition are currently experienced by young Brazilian persons, mediated by different social and historical realities, such as differences of class and gender^20^. In this way, it is important to identify and contextualize the different ways of transition from youth to adulthood, especially in a society marked by social inequality, such as the Brazilian society. Among the many possibilities to analyze this process, this article focuses on a particular group: young persons aged up to 19 years who are mothers.

The birth of a child is an important milestone in the transition to adulthood, but how has this fact fit into the lives of these young persons? Was it really the final milestone for the transition? How were work and school incorporated into the daily life of these young persons? Have they formed a new nuclear family or has the family of origin been extended? What is the pattern of the marital union? The answers to this series of questions can identify different sequences and forms in the process of transition to adulthood.

From these findings, this article analyzes the socio-demographic characteristics and the characteristics of sexual and reproductive behavior of young mothers, users of public health services, and the factors associated with the milestones of life stories that characterize the transition to adulthood.

The purpose of this study is to contribute to improving the data on adolescent pregnancy so that public policies can be established comprising the reproductive and social rights of this population group, considering their heterogeneity.

## METHODS

This is a cross-sectional study carried in Ribeirão Preto, Brazil. Ribeirão Preto is the most populous municipality of the State of São Paulo, with 649,556 inhabitants. Its Municipal Human Development Index (MHDI) of 0.800 is considered high by the United Nations Development Programme (UNDP). We have chosen this municipality exactly because of its good social conditions and low rates of pregnancy in mothers under the age of 19 years in relation to the State of São Paulo and other municipalities of the State with more than 100,000 inhabitants^22^.

We have interviewed 200 pregnant adolescents, with gestational age from the 36th week or recent mothers who used the services of the Brazilian Unified Health System (SUS) in the period from August to October, 2009, in Ribeirão Preto. We have included the three main maternity hospitals that users of the SUS go in the municipality; among them we mention the university service, which is a reference for high-risk prenatal care. We contacted all maternity hospitals and they agreed to participate in the study, giving us the access to hospitalized patients and outpatients. The interviewers attended all scheduled clinic hours. In addition, the birth records were checked daily to search for recent adolescent mothers.

We considered as eligible all adolescents hospitalized for childbirth or those who went to the maternity hospital for prenatal care during this period; all were invited to participate in the study. The face-to-face interviews were conducted by trained interviewers while the girls were waiting for their prenatal appointments, or when they were hospitalized after birth. There were no refusals.

The sample size for the estimation of proportions was set at 200, considering a population ratio of 0.50, sampling error of 10 percentage points, and 95% confidence level in the construction of confidence intervals.

This research is part of the Multicenter study “*Gestação na adolescência e uso do espaço urbano: vivências, expectativas e constituição de redes de apoio*”, supported by the CNPq. The applied questionnaire contained closed and semi-closed questions in a number of areas of interest to the project. For this article, we analyzed socio-demographic characteristics, family structure, education, sexual experience and life, and acceptance of pregnancy. We carried a pre-test with 36 young persons, with subsequent modification of the questions, mainly to adapt the questions and answers to the universe of the adolescents.

The independent variables analyzed were: age (categorized into 12 to 15 years and 16 to 19 years); self-reported race, according to methodology used in the Brazilian Census; economic class of the family using the *Critério de Classificação Econômica Brasil* (CCEB/ABEP – Brazilian Economic Classification Criterion – http//www.abep.org) that aggregates indicators for the schooling of the head of the family and possession of consumer goods; studying at the time of research; working in the year before pregnancy; age of beginning of sex life (categorized into up to 15 years and over 15 years); time with current partner, grouped into: less than a year, one to two years, and above two years; and having had another sex partner.

The dependent variables can be considered as markers of the transition from dependence to independence, from youth to adulthood. They are: planned pregnancy, living with a partner, and having left the parents’ house. We have considered planned pregnancy as an important indicator because it expresses the desire of the adolescent and it is not frequently used in studies with young mothers and fathers^8^. It is a way to explore the differences between pregnant adolescents, contributing to break the homogeneous vision of this phenomenon. The other two variables are also considered as milestones in the transition to adult life by several authors^7^ ^,^ ^12^.

The semi-closed answers were grouped by similarity to enable their analysis. We used the Shapiro-Wilk normality test. The quantitative variables were described using medians and minimum and maximum values, and the quality variables were described using absolute and relative frequencies. We also carried out a correlation analysis between the age of first sexual intercourse and age of current pregnancy. In the analysis of the associated factors, we used the Poisson model with robust variance. In the multiple model, we included all the variables that presented p < 0.20 in the bivariate analysis. The level of significance adopted was 5%.

For the bivariate and multiple model, the variable ‘race’ was reorganized in the categories “white” and “non-white” and the economic class was grouped into the classes “A or B”, “C” and “D or E”.

The face-to-face interviews were conducted by trained interviewers in privacy and confidentiality after the signing of the informed consent. This project has been approved by the Research Ethics Committee of the *Instituto de Ensino e Pesquisa Armênio Crestana* (Process 03/2008) and the *Hospital Universitário de Ribeirão Preto* of the *Universidade de São Paulo*.

## RESULTS

Average age was 17.3 years (SD = 1.57); 18.5% were aged between 13 and 15 years. Regarding race, most adolescents reported being brown (41.5%), followed by white (36.5%). The predominant economic class of the families of these young persons was C. Most (68%) lived with a partner and 10% were legally married (Table 1).

**Table 1:** Frequencies and proportions of the socio-demographic characteristics of the interviewees. Ribeirão Preto, State of São Paulo, Southeastern Brazil, 2009.

Characteristic	n	%
Age (years)		
13-15	37	18.5
16-17	69	34.5
18-19	94	47.0
Race		
White	73	36.5
Black	32	16.0
Brown	83	41.5
Other	12	6.0
Economic class*		
A or B	34	17.0
C	137	68.5
D or E	29	14.5
Marital status		
Single	180	90.0
Married	20	10.0
Marital status (n = 199)		
Lives with partner	136	68.0
Has a boyfriend, but does not live together	37	18.5
Single	26	13.0
Study		
Yes	38	19.0
Stopped before getting pregnant	97	48.5
Stopped in previous pregnancy	11	5.5
Stopped in the current pregnancy	54	27.0
Work before pregnancy		
No	97	48.5
Informal employment	97	48.5
Formal employment	6	3.0
Education level (years)		
0-4	3	1.5
5-8	90	45.0
≥ 9	107	53.5

* According to the *Critério de Classificação Econômica Brasil* 2013 (ABEP – Brazilian Economic Classification Criterion). Available from: http//www.abep.org

Regarding education, more than half had nine or more years of study; however, 21.0% had completed high school and 0.5% of the adolescents were undergraduates. Thirty-eight (19%) of the adolescents were studying at the time of research. Approximately 48.5% of them had dropped out of school before pregnancy (Table 1). Most of those who did not study anymore had finished high school. In the latter case, the statement “I have finished my studies” was the preponderant answer when asked why they were not studying. Of the young persons who were still studying, a little more than half lived with their parents. Approximately 48.5% of the adolescents had informal employment in occupations that demand low professional qualification (nannies, employed in trade, among others) in the year before their pregnancy (Table 1).

At the time of the interview, 143 young persons (71.5%) were in the immediate postpartum period and the other adolescents were in the last weeks of pregnancy. The average age of first sexual intercourse was 14.6 years, correlated positively (r = 0.54) with age in the current pregnancy (p < 0.01).

Talks about bodily changes and sexuality were reported by 135 of the adolescents (67.5%), most with family members; school and health services were the least mentioned places (6.5% and 2.5%, respectively). Adolescent pregnancy of other women in the family was mentioned by 74.5% of the interviewees.

Approximately half got pregnant from their first partner; the median time of relationship with the father of the baby was 12 months and approximately 1/4 of the adolescents were in a relationship with the same partner for more than two years (Table 2).

**Table 2:** Frequencies and proportions of the characteristics of sex life and pregnancy. Ribeirão Preto, State of São Paulo, Southeastern Brazil, 2009.

Characteristic	n	%
First time pregnancy	173	86.5
Age of the first intercourse (years)		
≤ 12	16	8.0
13-15	116	58.0
≥ 16	68	32.0
Family history of pregnant adolescents	149	74.5
Contraceptive methods		
Use of contraceptive method	73	36.5
Knowledge about the morning-after pill	135	67.5
Prior use of the morning-after pill	67	33.5
Time with the father of the baby (years)		
≤ 1	71	35.5
1-2	80	40.0
≥ 2	49	24.5
Number of sex partners		
1	102	51.0
2	37	18.5
3	28	14.0
≥ 4	33	16.5
Desire to get pregnant	63	31.5
Planning for pregnancy	50	25.0
Conversations about sexuality and bodily changes		
Yes	135	67.5
Reaction to the pregnancy news		
Happiness	88	44.0
Negative reactions (fear, sadness)	71	35.5
Surprise	39	19.5

For most, this was their first child. The pregnancy was planned by 25% of the interviewees. Regardless of the planning, most of the adolescents reported happiness with the pregnancy news, followed by negative reactions such as nervousness, fear, sadness and 19.7% reported surprise (Table 2). Of the total, 2% of the adolescents thought about aborting.

Approximately 1/3 of them used some contraceptive method when they got pregnant, although with several reports of irregular use. Among these, 70.3% used hormonal contraceptive methods, 28.4% used the male condom, and 1.4% used the two methods combined. The morning-after pill was known by 67.5% of the young persons and was used by 33.5% of them.

In the bivariate analysis (Table 3), we can verify a positive association between being with a partner for more than two years (p < 0.001) and not be studying (p = 0.02) with planned pregnancy. After adjusting the variables, time with partner from one year and economic class C were associated positively with planned pregnancy. The time with a partner was expanded in this analysis, keeping greater magnitude with more than two years. The variable on the study, observed in the bivariate analysis, lost its significance and did not adjust the other variables, being removed from the final model (Table 3).

**Table 3:** Proportion and crude and adjusted prevalence ratio of factors associated with planned pregnancy among young persons aged from 12 to 19 years. Ribeirão Preto, State of São Paulo, Southeastern Brazil, 2009.

Variable	Planned pregnancy					

	Yes	No	Crude PR		Adjusted PR	
			
			PR	95%CI	PR	95%CI
Time with partner (years)^a^						
≤ 1	11.3	88.7	1		1	
1-2	23.8	76.3	2.11	0.9–4.5	2.18	1.0–4.6^b^
≥ 2	46.9	53.1	4.16	2.0–8.6^b^	4.16	2.0–8.5^b^
Study^a^						
Yes	10.5	89.5	1		-	
No	28.4	71.6	2.7	1.0–7.0b	-	-
Age group						
12-15	13.5	86.5	1		-	
16-19	27.6	72.4	2.04	0.9–4.8	-	-
Economic class*						
A or B	11.8	88.2	1		1	
C	28.5	71.5	2.42	0.9–6.3	2.59	1.0–6.7^b^
D or E	24.1	75.9	2.05	0.7–6.3	2.46	0.8–7.4
Work						
Yes	28.9	71.1	1		1	
No	21.4	78.6	0.74	0.4–1.2	0.77	0.5–1.2

* According to the *Critério de Classificação Econômica Brasil* 2013 (ABEP – Brazilian Economic Classification Criterion). Available from: http//www.abep.org

^a^ p < 0.05 in the differences between proportions.

^b^ p < 0.05 in the model for associated factors.

In relation to living with a partner, the variables associated positively in the bivariate analysis were: planned pregnancy, being with a partner for more than two years, being white, and not having had other partners (Table 4). In the multiple analysis of this outcome, the same variables observed in the bivariate analysis remained positively associated, with the exception of race, with the respective magnitude adjustments (Table 4).

**Table 4:** Proportion and crude and adjusted prevalence ratio of factors associated with living with partner among young persons aged from 12 to 19 years. Ribeirão Preto, State of São Paulo, Southeastern Brazil, 2009.

Variable	Living with partner					

	Yes	No	Crude PR		Adjusted PR	
			
	%	%	PR	95%CI	PR	95%CI
Planned pregnancy^a^						
No	84.0	16.0	1		1	
Yes	62.7	37.3	1.34	1.1–1.6^b^	1.24	1.0–1.5^b^
Time with partner (years)^a^						
≤ 1	56.3	43.7	1		1	
1-2	68.8	31.2	1.22	0.9–1.6	1.18	0.9–1.5
≥ 2	83.7	16.3	1.48	1.2–1.9^b^	1.32	1.0–1.7^b^
Other partners^a^						
Yes	59.4	40.6	1		1	
No	75.5	24.5	1.27	1.0–1.6^b^	1.22	1.0–1.5^b^
Study^a^						
Yes	52.6	47.4	1		-	
No	71.6	28.4	1.36	0.9–1.9	-	-
Race^a^						
White	76.7	23.3	1		-	
Non-white	63.0	37.0	0.82	0.7–0.9	-	-
Age group (years)						
12-15	56.8	43.2	1		-	
16-19	70.6	29.4	1.24	0.9–1.7	-	-

^a^ p < 0.05 in the differences between proportions.

^b^ p < 0.05 in the model for associated factors.

Regarding leaving the parents’ house, in the bivariate analysis we observed a positive association with: planned pregnancy, economic class C, being with a partner for more than two years, not having had other partners, and not be studying (Table 5). In the multiple analysis, not having had another partner and not be studying lost statistical significance. Thus, the variables that continued to be positively associated with leaving the parents’ house were: planned pregnancy, economy class C, and being with a partner for more than two years (Table 5).

**Table 5:** Proportion and crude and adjusted prevalence ratio of factors associated with leaving the parents’ house among young persons aged from 12 to 19 years. Ribeirão Preto, State of São Paulo, Southeastern Brazil, 2009.

Variable	Leaving the parents’ house					

	Yes	No	Crude PR		Adjusted PR	
			
	%	%	PR	95%CI	PR	95%CI
Planned pregnancy^a^						
No	66.0	40.0	1		1	
Yes	33.3	66.7	1.8	1.3–2.5^b^	1.41	1.0–2.0^b^
Economic class*						
A or B	14.7	85.3	1		1	
C	48.2	51.8	3.27	1.4–7.5^b^	3.1	1.3–7.0^b^
D or E	31.0	69.0	2.11	0.8–5.6	2.05	0.8–5.2
Time with partner (years)^a^						
≤ 1	31.0	69.0	1		1	
1-2	37.5	62.5	1.21	0.8–1.9	1.19	0.8–1.8
≥ 2	57.1	42.9	1.84	1.2–2.8^b^	1.63	1.0–2.5^b^
Other partners^a^						
Yes	31.3	68.8	1		-	
No	47.1	52.9	1.5	1.0–2.2^b^	-	-
Study^a^						
Yes	23.7	76.3	1		-	
No	43.8	56.2	1.85	1.0–3.4^b^	-	-
Age group (years)						
12-15	32.4	67.6	1		-	
16-19	41.7	58.3	1.29	0.8–2.1	-	-

* According to the *Critério de Classificação Econômica Brasil* 2013 (ABEP – Brazilian Economic Classification Criterion). Available from: http//www.abep.org

^a^ p < 0.05 in the differences between proportions.

^b^ p < 0.05 in the model for associated factors.

## DISCUSSION

In relation to sampling, we must consider the fact that the sample was not drawn, as we have asked to answer the questionnaire all the adolescents who went to SUS for health services (pre-natal appointment and childbirth) in a period of three months. In the stage of data analysis, this set of adolescents was taken as a sample of adolescents cared for in 2009, based on the assumption that the outcomes studied do not suffer interference from the month of childbirth. Therefore, inferences were made for the population of adolescents, users of the SUS in the municipality of Ribeirão Preto, who have had their children that year. We highlight that the total number of births in the period, including the maternity hospitals of the private sector, was 1,037. Furthermore, this procedure assures comparability with other studies on adolescent pregnancy.

Changes in the reproductive and sexual profile of populations are, in general, generational processes. In this way, the collection of data in 2009 does not imply a limitation of the results presented herein. Additionally, in the same municipality, we have observed stability in the percentage of adolescents who gave birth between 2009 and 2013^a^.

^a^ DATASUS - Departamento de Informática do Sistema Único de Saúde. Brasília (DF); c2016 [cited 2014 Jun 30].Available from: http://www2.datasus.gov.br/DATASUS/index.php

The sociodemographic profile of the young persons interviewed have some particularities in relation to the results of research studies conducted in major Brazilian capitals^1^ ^,^ ^19^ and municipalities of similar size in the State of São Paulo^3^. The predominance of brown followed by white adolescents, with abandonment of school life, and higher entry into the labor market, although informally, before pregnancy, can be explained by some of the characteristics of the socio-economic development of the region, in which the modernization of farming caused changes both in the rural area and in the development of other economic sectors^16^. This process, on several occasions, was based on the precariousness of labor relations, both in the field and in cities^15^. If, on the one hand, Ribeirão Preto is a pole of attraction of skilled labor, on the other hand there is a growing space for informal employment. These young persons, with low schooling, enter the labor market, but informally and in fairly disqualified positions. We can assume that, with this profile of inclusion, they may not envision a professional growth among the possibilities for the future.

Although in this study we have interviewed only the adolescents who have chosen to keep the pregnancy, we note that only four of them reported having thought about the possibility of an abortion. Bell et al.^2^ have observed that, in a country where abortion is legal, young persons with more plans for the future, especially focusing on work and career, tend to choose abortion more.

The adolescents in this study come from families in which adolescent pregnancy is a common experience, a fact reported often in the literature in different realities^16^. The average age of first sexual intercourse (14.6 years) was similar to the results found by Doreto and Vieira^14^, in research carried out with the same type of population in the same city. The age of pregnancy was associated with the age of the first relationship, as verified in other realities^1^ ^,^ ^24^. This fact appears in several studies associated with lower schooling and lower purchasing power.

The use of contraceptive methods is similar to that observed in Brazilian capitals^1^ ^,^ ^19^. We highlight the report of condom use by only 11% of the partners, suggesting that the idea that contraception is the sole responsibility of women is common among young persons^10^. The apparent irregular use or non-use of these methods can be understood by them not announcing the start of their sex lives and the management of their sexuality to their families^9^. Furthermore, the beginning of the sexual activity also involves the negotiations of gender and the difficulties in the use of these methods^9^. Although most of them know about the morning-after pill, only a third had already resorted to that method.

The most positive responses in relation to being pregnant shows that this event can be greatly desired, even when it is not planned. In the case of Latin societies in general and the Brazilian society in particular, we can observe that motherhood has a central role in the life and appreciation of woman in societies^5^ ^,^ ^12^ ^,^ ^23^ and it falls on a change of social status, filled with positive significance, such as the establishment of new social networks. In the United States, one of every seven sexually active adolescent expresses positive attitudes in relation to pregnancy, and this proportion is greater in young persons from the most excluded strata of society^21^.

We note that more than half of the interviewees had sexual intercourse with only one partner. If on the one hand this suggests an agreement with the changes in sexual behavior in Brazil and in other Western societies, where sex initiation can occur before marriage^20^, on the other hand, this expresses a clear genre pattern, in which women tend to have fewer partners. For these young persons, this is more evident when they get pregnant from their first partner, often in long-lasting relationships, considering their ages^20^.

Heilborn et al.^19^ have identified three major groups of affective-sexual trajectory among young persons, from 18 to 24 years, living in Brazilian metropolises: young persons with stable relationship with one or at most two partners, young persons with various stable relationships with different partners, and young persons with no history of stable relationships. Despite the difference of the age group analyzed, we can identify that the portion of young mothers interviewed belongs to the first group. For them, the stable relationship with a partner is a common denominator in the execution of the various milestones of the transition from dependence to independence analyzed here (planning of pregnancy, marital life, and creation of a nuclear family).

The young persons of the popular universe tend to be often asked to assume roles restricted to home and child, especially if they live with a partner^12^ ^,^ ^18^. We identify here a pattern of male guardianship which leads to the entrance of the adolescent in the adult universe, as a mother and housewife. The high percentage of abandonment of employment after pregnancy confirms this trend. This path indicates that, for these young persons, pregnancy is not a result of casual dating, but an expected path, with cumulative and non-simultaneous situations^19^. Consequently, we can observe a high percentage of positive reactions to the pregnancy news. It is the exclusivity, the only partner, who identifies them as girls “who are to be married,” resuming the traditional concepts of education for women present in Brazil until the 1940s (respect, obedience, honesty, hard work, humility, gentleness, purity, ability to donate, domestic skills, and manual skills)^6^. The results found here suggest that the sequential pattern of transition from youth to adulthood can be assumed as an explanatory model for this first group. The changing role of women in society observed in recent decades, which means choosing a professional career, defining the number of children, and choosing their partner(s), has not reached this group of young persons.

Another group of adolescents, even after becoming mothers, keeps in their life history traces of dependence on the family of origin. For these young persons, the sequential model of transition is certainly not the most appropriate. As shown by Galland^17^, the birth of the child does not suggest the end of the transition.

We have identified the existence of distinct patterns of transition from youth to adulthood from different milestones of life examined (planning of pregnancy, living with partner, and leaving the parents’ house) in young mothers living in a municipality in the State of São Paulo, Brazil. Camarano^12^, analyzing the Brazilian youth group, has also observed the coexistence of sequential and non-sequential patterns of transition.

Public youth policies in general, and particularly for young mothers, must consider the differences in the life stories of these adolescents. As an example, income generation is as important policy for the group who has already started adulthood, while school life can still be central to the other groups. We need to ensure the expansion of the range of “possible futures” to all Brazilian young persons for the construction of a more just and fair society.
